# Knowledge, attitudes, perception and practices regarding antiretroviral therapy among HIV-infected adults in Antananarivo, Madagascar: a cross-sectional survey

**DOI:** 10.1186/s12913-019-4173-3

**Published:** 2019-05-28

**Authors:** Mihaja Raberahona, Zinara Lidamahasolo, Johary Andriamamonjisoa, Volatiana Andriananja, Radonirina Lazasoa Andrianasolo, Rivonirina Andry Rakotoarivelo, Mamy Jean de Dieu Randria

**Affiliations:** 1 Infectious Diseases Department, University Hospital Joseph Raseta Befelatanana, Antananarivo, Madagascar; 2 Department of Pharmacy, Faculty of Medicine, University of Antananarivo, Antananarivo, Madagascar; 3 Faculty of Medicine, University of Antananarivo, Antananarivo, Madagascar; 4 Infectious Diseases Department, University Hospital Tambohobe, Fianarantsoa, Madagascar

**Keywords:** Antiretroviral therapy, Knowledge, Attitude, Perception, Practices

## Abstract

**Background:**

Adherence to antiretroviral therapy (ART) may be influenced by knowledge, perception and perception regarding ART. The purpose of this study was to assess knowledge, attitude/perception and practice regarding ART among people living with HIV/AIDS (PLHIV).

**Methods:**

We conducted a cross-sectional survey to assess knowledge, attitudes, perception and practices ART in PLHIV. The survey was suggested to all PLHIV of at least 18 years old and who were on ART for at least 1 month. PLHIV who were unable to answer questions correctly and those who did not complete the survey for any reason were excluded.

**Results:**

During the study period, 234 PLHIV were included. Participants were mostly men (75.2%). The median age was 33 years (IQR: 27–41). The median time since HIV diagnosis was 25 months (IQR: 9–56) and the median duration of ART was 18 months (IQR: 8–48). 87.6% had an overall good knowledge of ART. However, only 3.2% knew the name of their ART, 31.2% were aware that ART should be taken at a fixed time and 17.1% knew how to take ART in relation to food intake. 75.6% of participants had an overall positive attitude/perception of ART. However, 10.7% were convinced that other methods were more effective than ART for treating HIV and 42.7% thought that taking ART was shameful. The assessment of practices showed that in case of missed dose, 48.3% of participants routinely skipped this dose instead of trying to take it as soon as possible. In multivariate analysis, good knowledge of ART was independently associated with high level of education (aOR: 4.7, IC95%: 1.6–13.7, *p* = 0.004) and disclosure of HIV status (aOR: 2.7, IC95%: 1.1–6.6, *p* = 0.029).

**Conclusions:**

This study showed an overall good knowledge and a predominantly positive attitude/perception of ART. However, accurate knowledge of ART intake was insufficient and the stigma associated with taking ART remained very present. Furthermore, very heterogeneous practices may reflect lack of instruction given by the physician regarding ART intake.

**Electronic supplementary material:**

The online version of this article (10.1186/s12913-019-4173-3) contains supplementary material, which is available to authorized users.

## Background

In 2016, the Joint United Nations Programme on HIV/AIDS (UNAIDS) estimated that 36.7 million people were living with HIV/AIDS including 19.5 million who were accessing antiretroviral therapy (ART). The African region remains the most affected region with 19.4 people living with HIV/AIDS (PLHIV) including 11.4 million PLHIV accessing ART.

Madagascar remains with a very low HIV prevalence of 0.2% compared to other African countries and 31,000 estimated PLHIV [1, 2]. As of October 2017, 2546 PLHIV were registered and 2026 have received ART (data from “Direction de lutte contre les IST/SIDA”, Ministry of Public Health). In 2016, Madagascar adhered to the UNAIDS Fast-track approach to end AIDS epidemic by 2030. This approach is a timeline that includes ambitious objectives and accelerating the delivery of HIV prevention and treatment services. One of the objectives of this approach is to achieve the 90–90-90 targets by 2020 with 90% of PLHIV knowing their status, 90% of people who know their status being treated and 90% of people receiving ART having a suppressed viral load. This target will be brought to 95–95-95 by 2030 [3].

ART substantially has improved survival of HIV-infected patient since the availability of highly active combination therapy in high-income countries as well as in middle- and low-income countries [4–6]. Since 2005, ART are available free of charge in Madagascar through a national program of care for PLHIV with the support and financing from the Global Fund. However, retention in care, non-adherence and ART attrition are recognized as challenges for PLHIV care programs in Africa including Madagascar [7–11].

Adherence to ART may be influenced by several factors including sociodemographic, economic, patient-related, treatment-related factors and problems related to medical services [9, 12–15]. Otherwise, several studies highlighted relationship between non-adherence to ART and lack of knowledge, negative perception and perception regarding ART. A study in Nepal has shown that PLHIV who thought that HIV could disappear after ART or those who thought that ART can be discontinued once they felt better had respectively 6.82 and 6.43 times more likely to be non-adherent. A meta-analysis including 207 studies has shown that perception about the utility of ART were strongly associated with adherence (standardized mean difference of 0.357, *p* = 0.001). PLHIV who knew their ART line of treatment and who were aware of the limited number of the therapeutic option were more likely to be adherent (adjusted prevalence ratio of 1.1; 95%CI: 1.0–1.3) [16–20]. Educational intervention remains a fundamental strategy in the management of PLHIV and helps improve adherence to ART [21–23]. However, educational intervention is a part of a comprehensive care including multiple and complex interventions.

We hypothesized that the assessment of knowledge, attitude and perception and practice regarding ART could help identify gaps that may be the target of an intensified educational intervention to improve adherence to ART.

This study aimed to assess knowledge, attitude, perception and practice regarding ART among HIV-infected patients.

## Methods

### Study design and setting

We conducted a cross-sectional survey for 2 months at the Infectious Diseases Department of the University Hospital Joseph Raseta Befelatanana, Antananarivo, Madagascar. This health facility is a tertiary referral hospital and the Infectious Diseases Department is a national referral center for the follow-up and hospitalization of HIV-infected patients especially those who are at AIDS stage with multiple opportunistic infections. This department also provide ART through a dedicated staff. ART is provided monthly to the patient. Patients have medical follow-up every three to 6 months. The public health system in Madagascar includes primary health care centers at community level (called CSB-1 and CSB-2). These centers provide basic health care including vaccination services, delivery services, maternal and child health care and management of common diseases. At district and regional level, there are intermediate level hospitals where cases from primary health care centers can be referred. These hospitals offer medical, basic surgical, laboratory and basic medical imaging facilities. Tertiary level hospitals offer a full range of care including specialized medical and surgical care, laboratory and complex imaging services. They also contribute to the training of the medical and paramedical staff in close collaboration with universities. All health care centers can provide HIV testing. ART are provided by trained medical staff in selected health care facilities including some primary health care centers, some hospitals at district or regional level or in some tertiary level hospitals.

### Inclusion and exclusion criteria

All PLHIV of at least 18 years old, who were on ART for at least 1 month and who gave a written consent were included in this study. Patients who were unable to answer because of neurological or psychiatric disorders and those who did not complete the interview for any reasons were excluded.

### Questionnaire

We carried out a review of the existing literature to develop and adapt a questionnaire assessing the knowledge, attitude and practice of ART in PLHIV as no standardized and validated questionnaire is available [10, 16, 19, 24–28] (Additional file 1).

We used open-ended questions to assess knowledge of ART for the following topics: ART drug name, ART dose, ART effect on HIV viral load and CD4 count and effect of missed doses of ART on treatment efficiency and the duration of ART. The answers to these questions were reviewed according to the actual ART regimen as documented in the medical record (ART drug name, ART dose and history of ART regimen) or according to the expected correct answer. For example, the answer to the question “How long should you take your ART” is expected to be “for lifetime”. Any other answers will be considered as false. We used categorical questions for the following topics: treatment schedule, way to take ART in relation to food intake (with food, without food, with or without food and on an empty stomach), history of ART regimen, purpose of ART, effect of missed dose on treatment efficiency and use of ART in the prevention of mother-to-child transmission. The recommendation regarding food intake was assessed according to the patient’s current ART regimen. Thus, the correct answer depends on the ART regimen. For example, the single-tablet regimen used as first line regimen (Tenofovir disoproxil/ Lamivudine/Efavirenz) should be taken on an empty stomach and this is considered to be the correct answer.

One point was assigned to each item assessing knowledge of ART which is correctly answered. A maximum score of 11 can be obtained. Lack of knowledge was defined by a score of 0 to 5. Good knowledge was defined by a score of 6 to 11.

We used dichotomous questions (Yes/No) to assess the attitude and perception of ART. Answers indicating a positive attitude/perception of ART were considered as expected correct answer. One point was assigned to each correct answer. For example, for the question “Are you really convinced to be infected by HIV”, the expected correct answer is “Yes”. A maximum score of six can be obtained. Negative attitudes and perception were defined by a score of 0 to 4. Positive attitudes and perception were defined by a score of 5 to 6.

A one-week pilot study was conducted on 10 participants. The questionnaire was modified according to the result of this pilot study. Questions that were difficult to understand or ambiguous or led to inappropriate or unexpected answers were modified accordingly.

### Data collection procedure

Participation to the study was proposed to all PLHIV who came to take ART and who met inclusion criteria. Participants were informed about the purpose of the study. Participants who finally signed a consent form were enrolled. Participants were interviewed by a trained investigator for about 10 to 20 min. Interview was done in Malagasy or in French or both at the discretion of the participant and the investigator. Data collected during the interview were noted in a de-identified form. Data collected through de-identified form were entered into a database using Epi-Info 7.2 (CDC, Atlanta, USA).

We collected in a semi-structured questionnaire (1) socio-demographic data including age, gender, sexual behavior, marital status, level of education, profession, belonging to an association; (2) data regarding ART; (3) data regarding knowledge of ART including drug name, dose, treatment schedule, effect of ART on viral load and CD4 count, effect of missed doses and use of ART in prevention of mother-to-child transmission; (4) attitude, perception regarding ART; (5) practice regarding ART including the choice of the storage, condition of storage, level of adherence, reasons of missed doses, use of reminder for ART, practice in case of missed doses, resources used to answer questions about ART and practice of self-medication.

### Statistical analysis

Continuous variables were described in median (interquartile range, IQR) and categorical variables were described in percentages. Continuous variables were compared using Mann-Whitney test and categorical variables were compared using Chi-square test or Fischer’s exact test as appropriate. A scoring system was used to define lack of knowledge/good knowledge and negative/positive attitude and perception about ART. A logistic regression model was used to determine independent factors associated with good knowledge of ART. Variables identified with a *P* value < 0.1 in univariate analysis were entered into the model. Associations were represented in odds-ratio (OR) and adjusted odds-ratio (aOR) with 95% confidence intervals (95%CI). A *P* value < 0.05 was considered as significant. Statistical analysis was performed using SPSS 23.0 (IBM Corp, Armonk, NY).

### Ethical considerations

All participants were informed about the purpose of the study and a written informed consent was obtained before enrolment. A verbal consent was obtained for illiterate participants and they were asked to provide a fingerprint on the consent form. In order to protect participants from unintentional disclosure of their HIV status, we did not ask to literate next of kin to provide written consent on behalf of illiterate participant. This study and the procedure used to obtain consent were approved by the National Ethics Committee of the Ministry of Public Health of Madagascar (N°087-MSANP/CERBM).

## Results

### Baseline characteristics

From September to October 2017, 260 PLHIV were invited to participate in an interview. Among them, 18 PLHIV refused to participate. The response rate was 93.1%. Eight PLHIV were excluded (3 PLHIV were unable to answer and 5 PLHIV did not complete the interview). A total of 234 PLHIV were included. Characteristics of PLHIV interviewed are detailed in Table 1. Participants were predominantly male. Median (IQR) age of male participants was lower than female participants: 32 years (IQR: 25–41) vs 34 years (IQR: 30–46), *p* = 0.008. Most of the participants were heterosexual (*n* = 138, 59%), single (*n* = 134, 57.3%), had high level of education (*n* = 115, 49.5%) and were currently employed (*n* = 141, 60.3%).

**Table 1:** Characteristics of PLHIV interviewed

Characteristics	n (%)
Age in years (median, IQR)	33 (27–41)
Male	176 (75.2)
Sexual orientation	
• Heterosexual	138 (59)
• Homosexual	35 (15)
• Bisexual	61 (26)
Marital status	
• Single	134 (57.3)
• Married	76 (32.5)
• Divorced	14 (6)
• Widowed	10 (4.3)
Educational level	
• Illiterate	2 (0.9)
• Primary	11 (4.7)
• Secondary	106 (45.3)
• Postgraduate	115 (49.5)
Currently employed	141 (60.3)
Student	44 (18.8)
Unemployed	49 (20.9)
Member of PLHIV association	31 (13.2)
Lives with other people	181 (77.4)
Number of people living under the same roof (median, IQR)	4 (3–5)
Disclosure of HIV status	163 (69.7)
Diagnosis of HIV in months (median, IQR)	25 (9–56)
• < 6 months	31 (13.2)
• 6–11 months	40 (17.1)
• 12–23 months	41 (17.5)
• 24–35 months	19 (8.1)
• ≥36 months	103 (44)
Duration of ART in months (median, IQR)	18 (8–48)
• < 6 months	37 (15.8)
• 6–11 months	46 (19.7)
• 12–23 months	40 (18.4)
• 24–35 months	28 (12.6)
• ≥36 months	80 (34.2)
ART regimen	
• TDF/3TC/EFV (1st line regimen)	212 (90.6)
• ABC/3TC/ATVr	15 (6.4)
• AZT/3TC/LPVr	2 (0.9)
• AZT/3TC/ATVr	1 (0.4)
• TDF/3TC/ATVr	2 (0.9)
• ABC/3TC/LPVr	2 (0.9)
Patient educational interventions	191 (81.6)

Among patients who have disclosed their HIV status, 56 (34.4%) have disclosed to their spouse, 36 (22.1%) to their mother, 21 (12.9%) to their father, 20 (12.3%) to their brother, 28 (17.2%) to their sister, 8 (4.9%) to their children, 28 (17.2%) to their friend, 28 (17.2%) to their sexual partner and 37 (22.7%) to people other than the medical staff. Among patients who have had educational interventions, 147 (77%) received educational interventions including education on HIV/AIDS and ART by referring physicians, 53 (27.7%) by ART dispensing staff, 23 (12%) by patient association, 11 (5.8%) by psychosocial support service and 22 (11.5%) by other stakeholders.

### Knowledge of ART

The assessment of knowledge of ART is detailed in Table 2. Median score for knowledge of ART was 7 (IQR: 6–8). Most of the participants exhibited a good knowledge (score ≥ 6) of ART (*n* = 205, 87.6%). However, only 9 participants (3.8%) were able to name their ART (brand name and/or name of all components of the ART regimen). Most of the participants knew that ART should be taken for lifetime. However, 25 participants gave other answers: ART should be taken until healing (*n* = 12), until the physician decided to stop it (*n* = 6), until viral load is undetectable (*n* = 4), during 6 months (*n* = 1), until CD4 count is equal or more than 500 per mm^3^ (*n* = 1) and for 4 years (*n* = 1).

**Table 2:** Knowledge of ART

	n (%)
What is the name of your ART?	
• Correct answer	9 (3.8)
• Incorrect answer	225 (96.2)
How many tablets should you take each day for your ART?	
• Correct answer	228 (97.4)
• Incorrect answer	6 (2.6)
How should you take your ART?	
• At fixed time^a^	73 (31.2)
• At variable time	161 (68.8)
How should you take your ART in relation to food intake?	
• Correct answer	40 (17.1)
• Incorrect answer	194 (82.9)
Has your ART regimen already been modified?	
• Correct answer	193 (82.5)
• Incorrect answer	41 (17.5)
How long should you take your ART?	
• Lifetime treatment^a^	195 (83.3)
• Other answer	25 (10.7)
• Don’t know	14 (6.0)
What is the purpose of ART?	
• Suppress the activity of HIV but do not cure^a^	214 (91.5)
• Cure HIV/AIDS	20 (8.5)
What is the effect of ART on HIV viral load?	
• Decrease HIV viral load^a^	169 (72.2)
• Other answer	44 (18.8)
• Don’t know	21 (9.0)
What is the effect of ART on CD4 count?	
• Increase CD4 count^a^	203 (86.8)
• Other answer	11 (4.7)
• Don’t know	20 (8.5)
What is the effect of missed doses on treatment efficiency?	
• Can reduce treatment efficiency^a^	204 (87.2)
• No effect	11 (4.7)
• Don’t know	19 (8.1)
Can ART prevent mother-to-child transmission of HIV?	
• Yes^a^	144 (61.5)
• No	60 (25.6)
• Don’t know	30 (12.8)

^a^ Expected correct answer

Participants who were not single have a significantly higher knowledge of the name of their ART than those who were single (7% vs 1.5%, *p* = 0.040). A significantly higher proportion of women were unaware of the dose of their ART compared to men (6.9% vs 1.1%, *p* = 0.035). The knowledge of treatment schedule was significantly higher in participants who were members of PLHIV associations (48.4% vs 28.6%, *p* = 0.027) and in participants who were not on first line ART regimen (68.2% vs 27.4%, *p* < 0.001). A significantly higher proportion of women were unaware of the history of their ART medication compared to men (29.3% vs 13.6%, *p* = 0.006). PLHIV who were diagnosed with HIV for less than 6 months (35% vs 11.2%, *p* = 0.008) or who were on ART for less than 6 months (35% vs 14%, *p* = 0.023) were significantly unaware of the purpose of ART compared to those who were not. Likewise, the proportion of PLHIV who were unaware of the purpose of ART was significantly lower in those who were single compared to those who were not (5.2% vs 13%, *p* = 0.035). The knowledge of the effect of ART on CD4 count was significantly higher in participants with a postgraduate level compared those with a lower level of education (92.2% vs 0%, *p* = 0.008). Similarly, the knowledge of the effect of ART on HIV viral load was higher in participants with a postgraduate level, however, this result did not reach statistical significance (76.5% vs 0%, *p* = 0.06). The knowledge of the preventive effect of the ART on mother-to-child transmission of HIV infection were significantly higher in PLHIV < 40 years (65.9% vs 50.7%, *p* = 0.032) and in PLHIV who do not live alone (65.2% vs 49.1%, *p* = 0.034).

In univariate analysis (Table 3), age < 40 years (OR: 2.7, 95%CI: 1.2–5.9, *p* = 0.012), postgraduate level (OR: 4.3, 95%CI: 1.7–11.1, *p* = 0.001), PLHIV currently employed and being student (OR: 2.7, 95%CI: 1.2–6.2, *p* = 0.016), disclosure of HIV status (OR: 2.4, 95%CI: 1.1–5.3, *p* = 0.025) and duration of ART≥6 months (OR: 2.8, 95%CI: 1.2–6.9, *p* = 0.016) were associated with good knowledge of ART.

**Table 3:** Factors associated with good knowledge of ART

Variables	Lack of knowledge (Score 0–5) n (%)	Good knowledge (Score 6–11) n (%)	Univariate analysis		Multivariate analysis	
			OR (95% CI)	*P-value*	aOR (95% CI)	*P-value*
Age < 40 years	15 (51.7)	152 (74.1)	2.7 (1.2–5.9)	0.012	1.9 (0.8–4.7)	0.147
Male	20 (69.0)	156 (76.1)	1.4 (0.6–3.3)	0.405		
Heterosexual	20 (69.0)	118 (57.6)	0.6 (0.3–1.4)	0.243		
Single	15 (51.7)	119 (58.0)	1.3 (0.6–2.8)	0.519		
Postgraduate level	6 (20.7)	109 (53.2)	4.3 (1.7–11.1)	0.001	4.7 (1.6–13.7)	0.004
Currently employed and student	18 (62.1)	167 (81.5)	2.7 (1.2–6.2)	0.016		
Member of PLHIV association	3 (10.3)	28 (13.7)	1.4 (0.4–4.8)	0.622		
Lives with other people	21 (72.4)	160 (78.0)	1.4 (0.6–3.3)	0.497		
Disclosure of HIV status	15 (51.7)	148 (72.2)	2.4 (1.1–5.3)	0.025	2.7 (1.1–6.6)	0.029
Patient educational interventions	24 (82.8)	167 (81.5)	0.9 (0.3–2.6)	0.866		
Diagnosis of HIV ≥ 6 months	22 (75.9)	181 (88.3)	2.4 (0.9–6.2)	0.065	0.5 (0.1–3.6)	0.464
Duration of ART ≥6 months	20 (69.0)	177 (86.3)	2.8 (1.2–6.9)	0.016	6.4 (0.9–43.3)	0.058
1st line ART regimen	29 (100.0)	183 (89.3)	–	0.085	–	0.998

*OR* odds-ratio, *aOR* adjusted odds-ratio, *95%CI* 95% confidence interval

In multivariate analysis, factors associated with good knowledge of ART (Table 3) were postgraduate level (aOR: 4.7, 95%CI: 1.6–13.7, *p* = 0.004) and disclosure of HIV status (aOR: 2.7, 95%CI: 1.1–6.6, *p* = 0.029).

### Attitude and perception of ART

The assessment of attitude and perception towards ART is detailed in Table 4. Median score for attitude and perception was 5 (IQR: 5–6). Most of the participants had a positive attitude and perception (score ≥ 5) towards ART (*n* = 177, 75.6%). Fifty-seven participants (24.4%) had negative attitude and perception. Among the 25 participants who believed in more effective method than ART for treating HIV, 10 participants refused to reveal the method they believed to be more effective than ART, 6 participants believed that religion is more effective, 5 participants believed that herbal medicine is more effective, 3 participants thought that there is more effective method than ART but they currently don’t know which one and 1 participant believed that healthy lifestyle is more effective than ART.

**Table 4:** Attitude and perception of ART

	n (%)
Do you believe that there are other more effective methods to treat HIV than ART?	
• Yes	25 (10.7)
• No^a^	209 (89.3)
Are you convinced of being infected by HIV?	
• Yes^a^	177 (75.6)
• No	57 (24.4)
Are you convinced of the effectiveness of ART?	
• Yes^a^	216 (92.3)
• No	18 (7.7)
Do you think that taking ART does more harm than good?	
• Yes	31 (13.2)
• No^a^	203 (86.8)
Are you convinced that you should continue your ART?	
• Yes^a^	229 (97.9)
• No	5 (2.1)
Do you feel ashamed to take your ART?	
• Yes	100 (42.7)
• No^a^	134 (57.3)

^a^ Expected correct answer

There were more participants who were convinced that there were other more effective methods than ART for treating HIV among those who were unemployed compared to those who were employed or student (18.4% vs 8.6%, *p* = 0.05). However, the result did not reach statistical significance. A significantly higher proportion of PLHIV with a diagnosis of HIV < 6 months were more convinced not being infected with HIV compared to those with a diagnosis of HIV ≥6 months (38.7% vs 22.2%, *p* = 0.046). In addition, a significantly higher proportion of PLHIV who were unemployed were convinced that ART does more harm than good compared to those who were employed or student (22.4% vs 10.8%, *p* = 0.033).

We did not identify factors associated with positive attitude and perception of ART in univariate and multivariate analysis.

### Practice regarding ART

Practice regarding ART was assessed and detailed in Table 5. We asked each participant for the choice of ART storage location at their home. One hundred forty-eight participants (63.2%) chose to store their medication “hidden and out of sight”, 77 participants (32.9%) chose a convenient storage but not necessarily suitable as recommended by the manufacturer, 55 participants (23.5%) chose a storage that can help to remember ART daily schedule, 45 participants (19.2%) chose a storage out of the reach and sight of children and 32 participants (13.7%) chose a suitable storage as recommended by the manufacturer. Most of the participants (*n* = 171, 73.1%) chose to store ART without its original carton packaging. In addition, 5 participants (2.1%) decided to store ART without its original plastic packaging and 11 participants (4.7%) stored ART in other plastic packaging.

**Table 5:** Practice regarding ART

	n (%)
Reasons for missed doses of ART (*n* = 151)	
• Can’t swallow pills	3 (2.0)
• Lack of information	1 (0.7)
• Busy doing something else	16 (10.6)
• Away from home	47 (31.1)
• Difficulty to comply with prescribed schedule	14 (9.3)
• Forgetting	56 (23.9)
• Health problem	8 (5.3)
• Too many tablets to take	3 (2.0)
• Other reasons	68 (45.0)
Method to remember to take ART	
• No particular method (habits)	190 (81.2)
• Help from a relative	28 (12.0)
• Reminder device	37 (15.8)
• Other	7 (3.0)
Practices in the event of missed dose of ART	
• Skip the missed dose and take the next dose planned	113 (48.3)
• Take the missed dose the next morning	74 (31.6)
• Call the referring physician	18 (7.7)
• Come to the hospital	14 (6.0)
• Take the missed dose as soon as possible	6 (2.6)
• Don’t know what to do	6 (2.6)
• Other	3 (1.2)
Resource used to answer questions regarding ART (*n* = 140)	
• Referring physician	81 (57.9)
• Internet	36 (25.7)
• Patient information leaflet	30 (21.4)
• ART dispensing staff	27 (19.3)
• Other PLHIV	13 (9.3)
• PLHIV association	8 (5.7)
• Do nothing	8 (5.7)
• Other people (including family member, friends, etc.…)	6 (4.3)
Practice of self-medication (*n* = 111)	
• Antibiotics	30 (27.0)
• Antipyretics	77 (69.4)
• NSAID	11 (9.9)
• Antimalarial drugs	4 (3.6)
• Proton pump inhibitor / H2 antagonists	5 (4.5)
• Decoction and herbal medicine	8 (7.2)
• Other	26 (23.4)

One hundred fifty-one participants (64.5%) reported that they have already missed doses of ART rarely (*n* = 139, 92.1%) or frequently (*n* = 12, 7.9%). During the last 7 days before the interview, 15 participants (6.8%) reported missed doses of ART: 1 dose for 12 participants (5.1%), 2 doses for 1 participant (0.4%) and 3 doses or more for 3 participants (1.3%). The main reasons for missed doses of ART were detailed in Table 5. Among the 68 participants (45%) who declared other reasons for missed doses of ART, 27 participants declared that they were travelling and did not have enough pills or they forgot to bring their medication, 12 participants had missed doses because they did not have time to take their medication at the hospital, 6 participants reported missed doses related to stock-outs of ART at the hospital, 3 participants had missed doses because they drank alcohol and wanted to avoid side effects, 3 participants had missed doses because they felt depressed, 2 participants have stopped ART to avoid side effects and 1 participant reported reminder device problem. 9 (3.8%) participants reported having already increased or decreased the dose of their ART without any prescription, 7 (3%) participants reported having already thrown their ART away and 13 (5.6%) reported having already lost their ART. The different practices of the participants in the event of missed doses are described in Table 5. Among the participants interviewed, 94 (40.2%) declared that they never had questions about their ART. The resources used by the other 140 participants (59.8%) to answer questions about their ART are detailed in Table 5. Among the 234 participants, 111 (47.4%) reported using of self-medication and only 35 participants (33%) felt that they were aware of the risk of the practice of self-medication with ART. Among participants who reported self-medicating, 55 (45%) relied on their own knowledge, 28 (25.2%) on past prescription, 23 (20.7%) on the advice of other people (excluding medical staff and pharmacists), 13 (11.7%) on the advice of pharmacists, 5 (4.5%) on the patient information leaflet and 2 (1.8%) on internet for self-medication issues.

## Discussion

This study highlighted an overall good knowledge of ART among PLHIV taking ART which is comparable to the level of knowledge in other studies [24, 25]. However, knowledge of some items was limited. Only 3.8% of the participants were able to give the name and/or International Non-proprietary Name of their ART. Patients often identified their treatment by the color of the tablets or the color of the drug box or the dose regimen. Otherwise, very few patients keep their prescription in practice. The real impact of this lack of knowledge may be limited as 97.4% of the participants were on first line regimen with fixed-dose triple-combination ART in once daily. However, a change in the presentation of the drug in case of change of supplier at the national program level which provided ART for PLHIV may lead to confusion for patients and should be avoided as much as possible. Irregular supply of ART is known as a major barrier to ART adherence [29]. However, the impact of change in drug presentation on adherence to ART is not actually predictable. Approximately four-fifths of the participants did not know recommendation regarding ART and food intake. Inadequate intake of ART with meals may result in an increased risk of side effects by increasing drug concentration as in efavirenz-based regimen or in decreased bioavailability as in Atazanavir-based regimen [30–32]. In our study, knowledge of dose was very high but two-third of the participants were unaware that ART should be taken at a fixed schedule. Recent study showed that patients were less respectful of dose schedule but highly adherent to dose [33]. Knowledge of ART dose could improve with time but it has been shown that poor knowledge 8 weeks after treatment initiation was predictive of poor adherence [34]. We found that high level of education was associated with good knowledge of ART. This high level of education could be explained by the location in the capital city of the health care facility where the survey was conducted and consequently raises concerns about knowledge of ART in remote areas were level of education is usually low. Disclosure of HIV status to a trusted person should be encouraged because of its influence on knowledge of ART as showed in our study but also because of its positive effect on adherence to ART and its impact on linkage and retention to care among PLHIV [35, 36].

Despite an overall positive attitude and perception of ART, a significant proportion of the participants were not convinced of the effectiveness of ART or thought that there is a more effective method than taking ART to treat HIV. These patients need to be closely monitored as they may be at high risk of discontinuing ART and loss to follow-up. Educational intervention as well as psychological support should be a continuous process in PLHIV to address retention in care issues. The stigma related to ART is still present among participants as 42.7% thought that taking ART was shameful. HIV treatment-related stigma was found to be much more significant in PLHIV compared to HIV-related stigma in general but has fortunately limited impact on HIV treatment uptake compared to other factors [37]. However, a negative attitude, perception or perception about ART has a negative impact on adherence to ART [16, 35].

In this study, only a small proportion of participants used reminder devices to improve ART intake. These strategies should be encouraged to improve adherence in addition to other interventions such as educational interventions or motivational groups support [21, 35]. However, the use of reminder devices may be limited in PLVIH who has not disclosed their HIV status. In this study, we also assessed practice in case of missed dose of ART. In fact, most of the participants never try to take the missed dose as soon as they remember but immediately decide to skip this dose and take the next one or take the missed dose the next morning. Most participants were on first-line ART regimen which is recommended to be taken once daily at bedtime. Therefore, a missed dose could be taken up to 12 h following the usual schedule. The practice in case of missed dose was assessed by open-ended questions and showed a large heterogeneity of the answers. Moreover, we previously noticed a lack of knowledge about timing and recommendation with respect to food intake for ART. These conditions may reflect lack of instructions from physicians on practical issues related to ART that should be reinforced.

Our study has several limitations. Answers to socio-demographic questions were based on participant’s declarations and could not be always checked. Moreover, some questions may not be fully understood or misinterpreted despite corrections and adaptations made during the pilot study as some medical terms could not be translated or are hard to translate in Malagasy and may have led to confusion. The sample size may have led to a lack of statistical power that could explain the inability of identifying factors associated to attitude and perception.

## Conclusion

Our study highlighted an overall good knowledge and positive attitude and perception of ART among participants. Knowledge and practice related to ART intake were substantially low and heterogeneous. Furthermore, stigma related to ART intake remained very high. The different gaps identified during this study should be targeted by specific interventions in PLHIV.

## Additional file

Additional file 1: Questionnaire used during the study. (DOCX 18 kb)

## Data Availability

The datasets used and/or analysed during the current study are available from the corresponding author on reasonable request.

## Abbreviations

3TC: Lamivudine
95%CI: 95% confidence interval
ABC: Abacavir
AIDS: Acquired Immune Deficiency Syndrome
aOR: Adjusted odds-ratio
ART: Antiretroviral therapy
ATVr: Atazanavir/ritonavir
AZT: Zidovudine
EFV: Efavirenz
HIV: Human Immunodeficiency Virus
IQR: Interquartile range
LPVr: Lopinavir/ritonavir
OR: Odds-ratio
PLHIV: People living with HIV/AIDS
TDF: Tenofovir
UNAIDS: Joint United Nations Programme on HIV/AIDS

## References

[1] UNAIDS Fact sheet July 2017. [http://www.unaids.org/sites/default/files/media_asset/UNAIDS_FactSheet_en.pdf]. Accessed 18 Aug 2018.

[2] Country factsheets Madagascar 2016. [http://www.unaids.org/fr/regionscountries/countries/madagascar]. Accessed 18 Aug 2018.

[3] Understanding fast-track - accelerating action to end the AIDS epidemic by 2030. [http://www.unaids.org/sites/default/files/media_asset/201506_JC2743_Understanding_FastTrack_en.pdf]. Accessed 23 Apr 2019.

[4] Antiretroviral Therapy Cohort C (2017). Survival of HIV-positive patients starting antiretroviral therapy between 1996 and 2013: a collaborative analysis of cohort studies. Lancet HIV.

[5] Slaymaker E, Todd J, Marston M, Calvert C, Michael D, Nakiyingi-Miiro J, Crampin A, Lutalo T, Herbst K, Zaba B (2014). How have ART treatment programmes changed the patterns of excess mortality in people living with HIV? Estimates from four countries in east and southern Africa. Glob Health Action.

[6] Reniers G, Slaymaker E, Nakiyingi-Miiro J, Nyamukapa C, Crampin AC, Herbst K, Urassa M, Otieno F, Gregson S, Sewe M (2014). Mortality trends in the era of antiretroviral therapy: evidence from the network for Analysing longitudinal population based HIV/AIDS data on Africa (ALPHA). AIDS.

[7] Rosen S, Fox MP, Gill CJ (2007). Patient retention in antiretroviral therapy programs in sub-Saharan Africa: a systematic review. PLoS Med.

[8] Fox MP, Rosen S (2010). Patient retention in antiretroviral therapy programs up to three years on treatment in sub-Saharan Africa, 2007-2009: systematic review. Tropical Med Int Health.

[9] Gesesew HA, Ward P, Woldemichael K, Mwanri L (2017). Prevalence, trend and risk factors for antiretroviral therapy discontinuation among HIV-infected adults in Ethiopia in 2003-2015. PLoS One.

[10] Nachega JB, Mills EJ, Schechter M (2010). Antiretroviral therapy adherence and retention in care in middle-income and low-income countries: current status of knowledge and research priorities. Curr Opin HIV AIDS.

[11] Kunutsor S, Walley J, Muchuro S, Katabira E, Balidawa H, Namagala E, Ikoona E (2012). Improving adherence to antiretroviral therapy in sub-Saharan African HIV-positive populations: an enhanced adherence package. AIDS Care.

[12] Wakibi SN, Ng'ang'a ZW, Mbugua GG (2011). Factors associated with non-adherence to highly active antiretroviral therapy in Nairobi, Kenya. AIDS Res Ther.

[13] Chesney MA (2000). Factors affecting adherence to antiretroviral therapy. Clin Infect Dis.

[14] Bezabhe WM, Chalmers L, Bereznicki LR, Peterson GM, Bimirew MA, Kassie DM (2014). Barriers and facilitators of adherence to antiretroviral drug therapy and retention in care among adult HIV-positive patients: a qualitative study from Ethiopia. PLoS One.

[15] Bijker R, Jiamsakul A, Kityo C, Kiertiburanakul S, Siwale M, Phanuphak P, Akanmu S, Chaiwarith R, Wit FW, Sim BL (2017). Adherence to antiretroviral therapy for HIV in sub-Saharan Africa and Asia: a comparative analysis of two regional cohorts. J Int AIDS Soc.

[16] Wasti SP, Simkhada P, Randall J, Freeman JV, van Teijlingen E (2012). Factors influencing adherence to antiretroviral treatment in Nepal: a mixed-methods study. PLoS One.

[17] Goujard C, Bernard N, Sohier N, Peyramond D, Lancon F, Chwalow J, Arnould B, Delfraissy JF (2003). Impact of a patient education program on adherence to HIV medication: a randomized clinical trial. J Acquir Immune Defic Syndr.

[18] Langebeek N, Gisolf EH, Reiss P, Vervoort SC, Hafsteinsdottir TB, Richter C, Sprangers MA, Nieuwkerk PT (2014). Predictors and correlates of adherence to combination antiretroviral therapy (ART) for chronic HIV infection: a meta-analysis. BMC Med.

[19] Nozaki I, Kuriyama M, Manyepa P, Zyambo MK, Kakimoto K, Barnighausen T (2013). False beliefs about ART effectiveness, side effects and the consequences of non-retention and non-adherence among ART patients in Livingstone, Zambia. AIDS Behav.

[20] Ramadhani HO, Muiruri C, Maro VP, Omondi M, Mushi JB, Lirhunde ES, Bartlett JA (2016). Association of knowledge on ART line of treatment, scarcity of treatment options and adherence. BMC Health Serv Res.

[21] Chaiyachati KH, Ogbuoji O, Price M, Suthar AB, Negussie EK, Barnighausen T (2014). Interventions to improve adherence to antiretroviral therapy: a rapid systematic review. AIDS.

[22] Kanters S, Park JJ, Chan K, Socias ME, Ford N, Forrest JI, Thorlund K, Nachega JB, Mills EJ (2017). Interventions to improve adherence to antiretroviral therapy: a systematic review and network meta-analysis. Lancet HIV.

[23] Rueda S, Park-Wyllie LY, Bayoumi AM, Tynan AM, Antoniou TA, Rourke SB, Glazier RH (2006). Patient support and education for promoting adherence to highly active antiretroviral therapy for HIV/AIDS. Cochrane Database Syst Rev.

[24] Olowookere SA, Fatiregun AA, Adewole IF (2012). Knowledge and attitudes regarding HIV/AIDS and antiretroviral therapy among patients at a Nigerian treatment clinic. J Infect Dev Ctries.

[25] Potchoo Y, Tchamdja K, Balogou A, Pitche VP, Guissou IP, Kassang EK (2010). Knowledge and adherence to antiretroviral therapy among adult people living with HIV/AIDS treated in the health care centers of the association "Espoir vie Togo" in Togo, West Africa. BMC Clin Pharmacol.

[26] Ramchandani SR, Mehta SH, Saple DG, Vaidya SB, Pandey VP, Vadrevu R, Rajasekaran S, Bhatia V, Chowdhary A, Bollinger RC (2007). Knowledge, attitudes, and practices of antiretroviral therapy among HIV-infected adults attending private and public clinics in India. AIDS Patient Care STDs.

[27] Dyrehave C, Rasmussen DN, Honge BL, Jespersen S, Correia FG, Medina C, Wejse C, Rodkjaer L, Bissau HIVCSG (2016). Nonadherence is associated with lack of HIV-related knowledge: a cross-sectional study among HIV-infected individuals in Guinea-Bissau. J Int Assoc Provid AIDS Care.

[28] Agnarson AM, Levira F, Masanja H, Ekstrom AM, Thorson A (2013). Antiretroviral treatment knowledge and stigma--implications for programs and HIV treatment interventions in rural Tanzanian populations. PLoS One.

[29] Mills EJ, Nachega JB, Bangsberg DR, Singh S, Rachlis B, Wu P, Wilson K, Buchan I, Gill CJ, Cooper C (2006). Adherence to HAART: a systematic review of developed and developing nation patient-reported barriers and facilitators. PLoS Med.

[30] Lamorde M, Byakika-Kibwika P, Tamale WS, Kiweewa F, Ryan M, Amara A, Tjia J, Back D, Khoo S, Boffito M (2012). Effect of food on the steady-state pharmacokinetics of Tenofovir and Emtricitabine plus Efavirenz in Ugandan adults. AIDS Res Treat.

[31] Colombo S, Buclin T, Cavassini M, Decosterd LA, Telenti A, Biollaz J, Csajka C (2006). Population pharmacokinetics of atazanavir in patients with human immunodeficiency virus infection. Antimicrob Agents Chemother.

[32] Goutelle S, Baudry T, Gagnieu MC, Boibieux A, Livrozet JM, Peyramond D, Chidiac C, Tod M, Ferry T, Lyon HIVCSG (2013). Pharmacokinetic-pharmacodynamic modeling of unboosted Atazanavir in a cohort of stable HIV-infected patients. Antimicrob Agents Chemother.

[33] Tiruneh YM, Wilson IB (2016). What time is it? Adherence to Antiretroviral Therapy in Ethiopia. AIDS Behav.

[34] Miller LG, Liu H, Hays RD, Golin CE, Ye Z, Beck CK, Kaplan AH, Wenger NS (2003). Knowledge of antiretroviral regimen dosing and adherence: a longitudinal study. Clin Infect Dis.

[35] Heestermans T, Browne JL, Aitken SC, Vervoort SC, Klipstein-Grobusch K (2016). Determinants of adherence to antiretroviral therapy among HIV-positive adults in sub-Saharan Africa: a systematic review. BMJ Glob Health.

[36] Ostermann J, Pence B, Whetten K, Yao J, Itemba D, Maro V, Reddy E, Thielman N (2015). HIV serostatus disclosure in the treatment cascade: evidence from northern Tanzania. AIDS Care.

[37] Cama E, Brener L, Slavin S, de Wit J (2015). The impact of HIV treatment-related stigma on uptake of antiretroviral therapy. AIDS Care.

