# Supplementary material for: Knowledge, attitudes, perception and practices regarding antiretroviral therapy among HIV-infected adults in Antananarivo, Madagascar: a cross-sectional survey
Source: BMC Health Serv Res. 2019 May 28;19:341. doi: 10.1186/s12913-019-4173-3 (PMC6537363; doi:10.1186/s12913-019-4173-3)
Supplement: Supplementary file 1 — Questionnaire used during the study. (DOCX 18 kb) [file 12913_2019_4173_MOESM1_ESM.docx]

**QUESTIONNAIRE**

| **SOCIO-DEMOGRAPHIC DATA** | |
| --- | --- |
| 1. **Date of inclusion** | [_D_][_D_]/[_M_][_M_]/[Y_][_Y_] |
| 1. **Identification** | [___][___][___][___][___][___] |
| 1. **Marital status** | ☐ Single ☐ Married ☐ Divorced ☐ Widowed |
| 1. **Sexual orientation** | ☐ Heterosexual ☐ Homosexual ☐ Bisexual |
| 1. **Profession** | ☐ Student ☐ Unemployed  ☐ Employed __________________________________ |
| 1. **Level of education** | ☐ Illiterate ☐ Primary  ☐ Secondary ☐ Postgraduate |
| 1. **Date of HIV diagnosis** | [_D_][_D_]/[_M_][_M_]/[Y_][_Y_] |
| 1. **Start date of ART** | [_D_][_D_]/[_M_][_M_]/[Y_][_Y_] |
| 1. **Association member** | ☐ Yes ☐ No |
| 1. **Lives alone** | ☐ Yes ☐ No |
| 1. **Number of people living under the same roof** | _______________________ |
| 1. **Educational interventions** | ☐ Yes ☐ No |
| **If yes by** | ☐ Referring physician ☐ ART dispensing staff  ☐ Patient association ☐ Psychosocial support service  ☐ Other |
| 1. **Disclosure of HIV** | ☐ Yes ☐ No |
| **If yes, disclosure to** | ☐ Spouse ☐ Mother ☐ Father ☐ Brother  ☐ Children ☐ Friends ☐ Sexual partner  ☐ Other than medical staff |
| **KNOWLEDGE OF ANTIRETROVIRAL THERAPY** | |
| 1. **What is the name of your ART?** | ______________________________________________ |
| 1. **How many tablets should you take each day for your ART?** | ______________________________________________ |
| 1. **How should you take your ART?** | ☐ At a fixed time ☐ At a variable time |
| 1. **How should you take your ART in relation to food intake** | ☐ With food ☐ Without food  ☐ Either with or without food  ☐ On an empty stomach |
| 1. **Has your ART regimen already been modified?** | ☐ Yes ☐ No |
| 1. **How long should you take your ART?** | _________________________________________ |
| 1. **What is the purpose of ART?** | ☐ Suppress the activity of HIV but do not cure ☐ Cure HIV/AIDS |
| 1. **What is the effect of ART on HIV viral load?** | _____________________________________________ |
| 1. **What is the effect of ART on CD4 count?** | _____________________________________________ |
| 1. **What is the effect of missed doses on treatment efficiency?** | _____________________________________________ |
| 1. **Can ART prevent mother-to-child transmission of HIV?** | ☐ Yes ☐ No |
| **ATTITUDE AND PERCEPTION OF ANTIRETROVIRAL THERAPY** | |
| 1. **Do you believe that there are other more effective methods to treat HIV than ART?** | ☐ Yes ☐ No |
| 1. **Are you convinced of being infected by HIV** | ☐ Yes ☐ No |
| 1. **Are you convinced of the effectiveness of ART** | ☐ Yes ☐ No |
| 1. **Do you think that taking ART does more harm than good** | ☐ Yes ☐ No |
| 1. **Are you convinced that you should continue your ART?** | ☐ Yes ☐ No |
| 1. **Do you feel ashamed to take your ART?** | ☐ Yes ☐ No |
|  |  |
| **PRACTICE REGARDING ANTIRETROVIRAL THERAPY** | |
| 1. **Where do you store your ART at home?** | ☐ Hidden and out of sight  ☐ Convenient storage but not necessarily as recommended by the manufacturer  ☐ Storage that can help to remember daily schedule  ☐ Storage out of the reach and sight of children  ☐ Suitable storage as recommended by the manufacturer |
| 1. **How do you store your ART at home?** | ☐ Without its original carton packaging  ☐ Without its original plastic packaging  ☐ In other plastic packaging  ☐ In its original carton and plastic packaging |
| 1. **Have you already missed a dose of ART?** | ☐ Yes ☐ No |
| **If yes** | ☐ Rarely ☐ Frequently |
| 1. **During the last 7 days, how many times have you missed taking your ART?** | _____________________________________________ |
| 1. **Why have you missed taking your ART?** | _____________________________________________ |
| 1. **What do you do if you have missed a dose of ART?** | __________________________________________________________ |
| 1. **How do you remember to take your ART?** | ☐ No particular method (habits)  ☐ Help from a relative  ☐ Reminder device  ☐ Other: __________________________________________________________ |
| 1. **Have you ever increased or decreased the dose of your ART?** | ☐ Yes ☐ No |
| 1. **Have you ever thrown away your ART?** | ☐ Yes ☐ No |
| 1. **Have you ever lost your ART?** | ☐ Yes ☐ No |
| 1. **Have you ever had questions about your ART?** | ☐ Yes ☐ No |
| 1. **Who do you ask or where do you find out information about your ART?** | _____________________________________________ |
| 1. **Have you ever practiced self-medication?** | ☐ Yes ☐ No |
| 1. **What type of drugs have you already taken in self-medication?** | _____________________________________________ |
| 1. **Where do you find out information when you are self-medicating?** | _____________________________________________ |
